# Investigation into the Simulation and Mechanisms of Metal–Organic Framework Membrane for Natural Gas Dehydration

**DOI:** 10.3390/nano14191583

**Published:** 2024-09-30

**Authors:** Qingxiang Song, Pengxiao Liu, Congjian Zhang, Yao Ning, Xingjian Pi, Ying Zhang

**Affiliations:** 1 College of Science, China University of Petroleum (Beijing), Beijing 102249, China; norm119@163.com; 2 PetroChina Tarim Oilfield Company, Kuerle City 841000, China; 3 Collaborative Innovation Center of Capital Resource-Recycling Material Technology, College of Materials Science and Engineering, Beijing University of Technology, Beijing 100124, China; 4 College of New Energy and Materials, China University of Petroleum (Beijing), Beijing 102249, China

**Keywords:** natural gas dehydration, molecular dynamics simulation, metal–organic composite membrane, temperature, mechanism

## Abstract

Natural gas dehydration is a critical process in natural gas extraction and transportation, and the membrane separation method is the most suitable technology for gas dehydration. In this paper, based on molecular dynamics theory, we investigate the performance of a metal–organic composite membrane (ZIF-90 membrane) in natural gas dehydration. The paper elucidates the adsorption, diffusion, permeation, and separation mechanisms of water and methane with the ZIF-90 membrane, and clarifies the influence of temperature on gas separation. The results show that (1) the diffusion energy barrier and pore size are the primary factors in achieving the separation of water and methane. The diffusion energy barriers for the two molecules (CH_4_ and H_2_O) are ΔE(CH_4_) = 155.5 meV and ΔE(H_2_O) = 50.1 meV, respectively. (2) The ZIF-90 is more selective of H_2_O, which is mainly due to the strong interaction between the H_2_O molecule and the polar functional groups (such as aldehyde groups) within the ZIF-90. (3) A higher temperature accelerates the gas separation process. The higher the temperature is, the faster the separation process is. (4) The pore radius is identified as the intrinsic mechanism enabling the separation of water and methane in ZIF-90 membranes.

## 1. Introduction

Natural gas, as a clean fossil energy product, is and will remain an essential energy source now and in the future [1,2]. Raw natural gas contains a certain proportion of water vapor, which can condense into liquid water when the temperature in the natural gas pipeline falls below the water dew point. This liquid water, upon contact with acidic gases in natural gas (such as H_2_S and CO_2_), forms acids that accelerate the corrosion of pipelines and equipment. Therefore, natural gas dehydration is a crucial step in the extraction and transportation of natural gas [3,4,5,6,7]. In view of this, natural gas dehydration in natural gas extraction and transportation is important. Efficient dehydration technology prevents pipeline corrosion, reduces transportation costs, and improves the quality of natural gas. Currently, there are three main methods for natural gas dehydration: solvent absorption [8,9,10], solid adsorption [11,12,13,14], and membrane separation [15,16,17]. Research [18,19,20,21] has shown that membrane separation is the most suitable technology for natural gas dehydration. However, the traditional dehydration method has the problems of high energy consumption and low efficiency. Therefore, the development of high-performance membrane separation materials has important practical significance. The selection of membrane materials has become a research hotspot in the field of natural gas dehydration.

Membrane materials are at the core of gas membrane separation technology. Traditional single-component membrane materials can no longer meet the demands of usage, requiring higher performance standards for separation membranes. Among the many materials, composite materials have demonstrated significant superiority and have attracted considerable attention in gas separation.

Xu et al. [22] reported an interfacially bonded and highly efficient CO_2_ separation membrane, which was fabricated by blending UiO-66-MA nanoparticles with reactive allyl groups and Polyethylene oxide (PEO) macromer monomers with double bonds, followed by UV-induced free radical crosslinking to form the nanocomposite membrane. Ban et al. [23] proposed a method to customize the performance of cage-type (metal–organic framework) MOF molecular sieves by introducing cavity occupants to alter the microenvironment of MOF nanocages. Ban et al. [23] synthesized the MOF materials by dissolving zinc nitrate and 2-methylimidazole in 1-butyl-3-methylimidazolium bis(trifluoromethylsulfonyl) imide (an ionic liquid that serves as both solvent and cavity occupant).

The use of numerical simulations to accelerate the selection of separation membrane materials has become a trend. Numerical simulation aids in the design and development of materials, not only providing accurate flux trends for various gases but also offering reliable estimates of selectivity. Permeability and selectivity are crucial for the practical application of these new materials in separation processes in the foreseeable future. Modern computational tools can accelerate the development of two-dimensional porous materials for membrane applications.

Lin et al. [24] used numerical simulations to calculate the processes of four typical natural gas dehydration membrane modules and found that the counter-current design using dry gas flow as sweep gas is the most economical process for natural gas dehydration membrane modules. Jaramillo et al. [4] obtained adsorption isotherms of CO_2_, NH_3_, and H_2_O in 4A zeolite at different temperatures through simulations and identified the geometric shape of adsorption sites and their dependence on loading, with simulation isotherms closely matching experimental data. Eva et al. [7] studied the effect of different sodium and calcium cation compositions on the hydration process in alumina-rich LTA-type zeolite (Si/Al = 1) using molecular simulation and concluded that the influence of cation properties is much greater than that of cation density.

**Figure 1:**
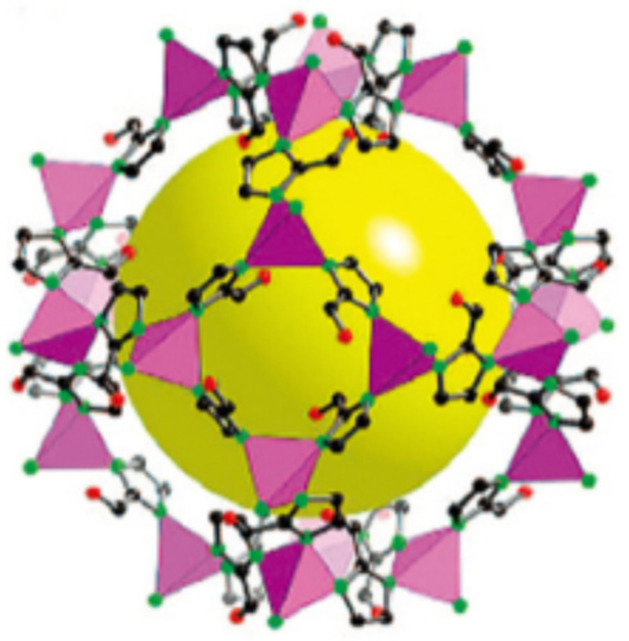
Structure of the ZIF-90. Purple: Zn; Red: O; Black: C; Green: N.

Moreover, Jiang et al. [25] investigated the influence of the pore size and functional group polarity on seawater desalination in zeolitic imidazolate frameworks (ZIFs, a type of MOF structure, as shown in Figure 1) using molecular simulation and tested the seawater desalination performance of ZIF membranes with different pore sizes (ZIF-8, -93, -95, -97, and -100) through reverse osmosis. Gao et al. [7] studied the effects of van der Waals forces and electrostatic forces on water adsorption capacity. By synthesizing a series of seven ZIF materials with different pore sizes (ZIF-8, ZIF-90, SIM-1, MAF-6, ZIF-25, ZIF-93, and ZIF-97) and combining experimental and computational methods, they concluded that van der Waals interactions can be neglected, and electrostatic interactions dominate. Fredy et al. [26] analyzed the dynamic behavior of CO_2_/N_2_ and CO_2_/CH_4_ gas mixtures in IRMOF-1 and Cu-BTC membranes with the molecular dynamics simulations. Fredy et al. [26] proposed two methods for simulating membrane systems, constant pressure gradient permeation and variable pressure gradient permeation, and found that both materials have similar effects in separating CO_2_/N_2_, with Cu-BTC being most suitable for separating CO_2_/CH_4_ mixtures. Tony Pham et al. [27] used grand canonical Monte Carlo (GCMC) simulations to study the adsorption of CO_2_ and H_2_ by metal–organic frameworks (MOFs) synthesized from nitrogen-rich triazinyl and amine functional groups.

However, the above studies [22,23,24,25,26,27] indicate that research on composite materials for gas separation membranes is not comprehensive; it fails to reveal the intrinsic mechanisms of gas separation in natural gas at the molecular level and the effects of factors such as temperature and pressure on natural gas dehydration, as well as the diffusion behavior of water molecules in metal–organic framework membranes during natural gas dehydration.

Therefore, in this paper, we focus on the metal–organic framework ZIF-90, using molecular dynamics theory and simulation calculations to study the application of ZIF-90 membranes in gas separation. The research investigates the performance of natural gas dehydration under different temperature and pressure conditions, reveals the intrinsic mechanisms of methane/water vapor separation at the molecular level with the ZIF-90 membrane materials, clarifies the effects of temperature as well as pressure on gas diffusion and natural gas dehydration, and identifies the diffusion pathways of water molecules within the ZIF-90 membrane, providing valuable reference results for the development of natural gas dehydration.

## 2. Computational Model and Calculation Details

The initial unit cell structure of the ZIF-90 was constructed based on X-ray crystallography results. The space group of the ZIF-90 is I-43m, with lattice parameters a = b = c = 17.2715 Å, where each unit cell contains a cage with a diameter of 15 Å. The cage has openings formed by six-membered rings, each with a diameter of approximately 3.5 Å. The four-membered rings are too small for most gas molecules (H_2_O, CO_2_, CH_4_, N_2_, etc.) to pass through. The structure of the ZIF-90 [28] is shown in Figure 1, presenting a typical cage-shaped structure. In this paper, the ZIF-90 was used as the membrane material to investigate the gas diffusion and adsorption performance. The H_2_O as well as CH_4_ gas molecules were placed on one side of the ZIF-90 membrane along the z-axis.

All density functional theory (DFT)-based calculations were performed with the Materials Studio (MS 2020) software package. In order to optimize the structure and minimize the energy, the generalized gradient approximation (GGA) and the PBE method were employed. The cutoff energy for the plane-wave basis, the convergence criteria for intermolecular forces, as well as the energy, were set to 597.8 eV, 0.03 eV/Å, and 10^−5^ eV, respectively.

The energy barrier for gas molecules passing through the six-membered rings was calculated by varying the height of H_2_O and CH_4_ molecules relative to the plane of the six-membered rings. In order to obtain the free energy curve of the molecule, we set up several sampling windows along the axial direction of the molecule through the membrane pore channel, with an interval of 0.1 A. In each window, a harmonic potential with a force constant of 1000 kJ·mol^−1^·nm^−2^ was applied to restrict the molecule to a particular position. Each window ran for 2 ns for a total simulation time of 200 ns.

GCMC and MD simulations were also carried out using the MS software (2020), with all simulations conducted in the Canonical (NVT) ensemble with a time step of 1.0 fs. Meanwhile, the periodic boundary conditions were applied in all directions (x, y, and z) in this paper. In all MD simulations, the Coulombic and Lennard-Jones (LJ) potentials were used to describe the interactions between gas molecules. The system temperature was set at 298 K, the pressure was 1 atm, and the Nosé–Hoover thermostat and Parrinello–Rahman pressure coupling method were used.

The ZIF-90 model was kept fixed, and a cutoff distance of 9 Å was applied for pair-to-pair comparisons. The Coulombic and Lennard-Jones(LJ) potentials used to describe the interactions between gas molecules, whose potential parameters together with the charges [29] used, are provided in Table 1. The Three-Site Point Charge Model (TIP3P) potential was used for water (H_2_O), and the Transferable Potentials for Phase Equilibria (TraPPE) potential was used for methane (CH_4_). Furthermore, the Lorentz–Berthelot mixing rules were used to define the LJ interactions between different atoms.

## 3. Results and Discussion

### 3.1. Diffusion Energy Barriers

To obtain the free energy profile of the molecules, multiple sampling windows were set along the axial direction of the molecules passing through the membrane pores, with an interval of 0.1 Å. In each window, a harmonic potential with a force constant of 1000 kJ·mol^−1^·nm^−2^ was applied to constrain the molecules at specific positions. Each window was simulated for 2 ns, totaling 200 ns of simulation time.

**Figure 2:**
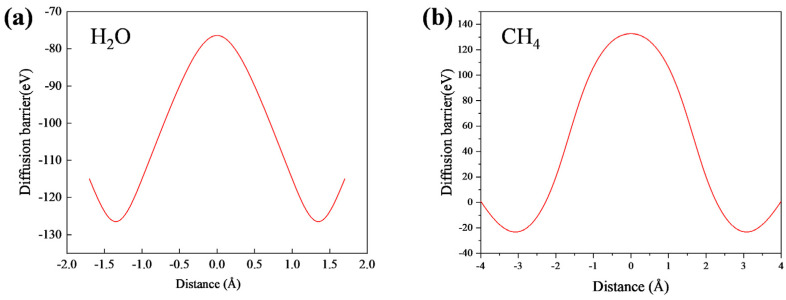
The diffusion energy barrier for (**a**) H_2_O and (**b**) CH_4_.

As shown in Figure 2a,b, the diffusion energy barriers for the two molecules (CH_4_ and H_2_O) are ΔE(CH_4_) = 155.5 meV and ΔE(H_2_O) = 50.1 meV, respectively. This indicates that the water (H_2_O) molecules experience a negligible energy barrier when the H_2_O molecules enter the pores, which is mainly due to the favorable interaction between the polar H_2_O molecules and the ZIF-90 membrane, as well as the smaller size of H_2_O. In contrast, CH_4_ encounters a higher energy barrier to enter the pores [30].

Methane molecules have a larger kinetic diameter (about 3.8 A), which is close to the ZIF-90 membrane pore size (about 3.5 A), and, thus, are spatially limited when passing through the pore channels, increasing the diffusion energy barrier. In addition, methane is a non-polar molecule that interacts weakly with the pore wall and lacks the interaction force that helps reduce the energy barrier. In contrast, water molecules are smaller in size (about 2.65 A) and more easily pass through pores. At the same time, water molecules form hydrogen bonds and coordinate with polar functional groups in the ZIF-90 membrane, such as aldehyde groups and Zn^2+^, and these favorable interactions reduce the diffusion energy barrier of water molecules.

### 3.2. Adsorption and Diffusion Performance

**Figure 3:**
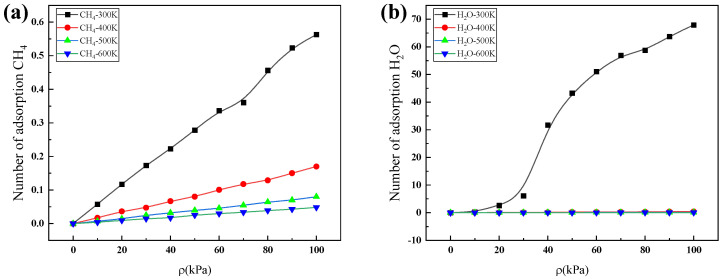
Adsorption isotherms for (**a**) CH_4_ and (**b**) H_2_O on ZIF-90 at different temperatures.

The adsorption performances of H_2_O and CH_4_ molecules in a ZIF-90 unit cell were calculated with the grand canonical Monte Carlo (GCMC) method, and their adsorption isotherms at different temperatures are shown in Figure 3. The simulated adsorption isotherm is consistent with the experimental results [31,32,33,34]. In Figure 3, it can be seen that a low temperature and a high pressure are conducive to the adsorption of gas molecules. The adsorption capacity of H_2_O molecules increases sharply with increasing pressure at low pressure and then tends towards a constant value, while the adsorption capacity of CH_4_ molecules increases linearly with increasing pressure. The adsorption capacity of H_2_O molecules is much greater than that of CH_4_ molecules, and the adsorption capacity of H_2_O molecules at room temperature and pressure is 120 times that of CH_4_ molecules. From this, it can be concluded that the ZIF-90 materials present strong adsorption performance for H_2_O molecules, but weak adsorption performance for CH_4_ molecules, indicating that the ZIF-90 has adsorption selectivity for separating strongly adsorbed gas H_2_O and weakly adsorbed gas CH_4_.

In order to quantitatively investigate the diffusion behavior of gas molecules within the ZIF-90 membrane, the diffusion coefficients of the gas molecules were calculated under different temperatures and pressures. For simple Brownian diffusion of gas molecules, the diffusion coefficient is represented by the Einstein Equation (1), which relates the atomic position r to the correlation function of the diffusion rate.

(1) $$D=\frac{1}{6}\underset{t\to \infty }{\lim}\frac{{\left[r\left(t\right)-r\left(0\right)\right]}^{2}}{t}$$

In Equation (1), t is the simulation time, and [r(t) − r(0)]^2^ is the mean square displacement (MSD). The diffusion coefficients at different temperatures as well as pressures were calculated using the best linear fit of the mean square displacement curves.

**Figure 4:**
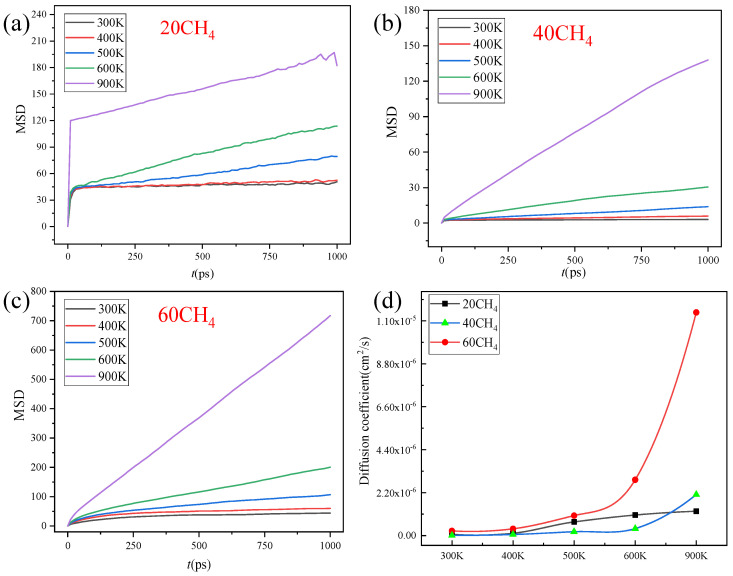
MSD (**a**–**c**) and diffusion coefficient (**d**) of CH_4_ on ZIF-90 at different temperatures. (**a**) 20 CH_4_ molecules, (**b**) 40 CH_4_ molecules, (**c**) 60 CH_4_ molecules. (**d**) The diffusion coefficient.

**Figure 5:**
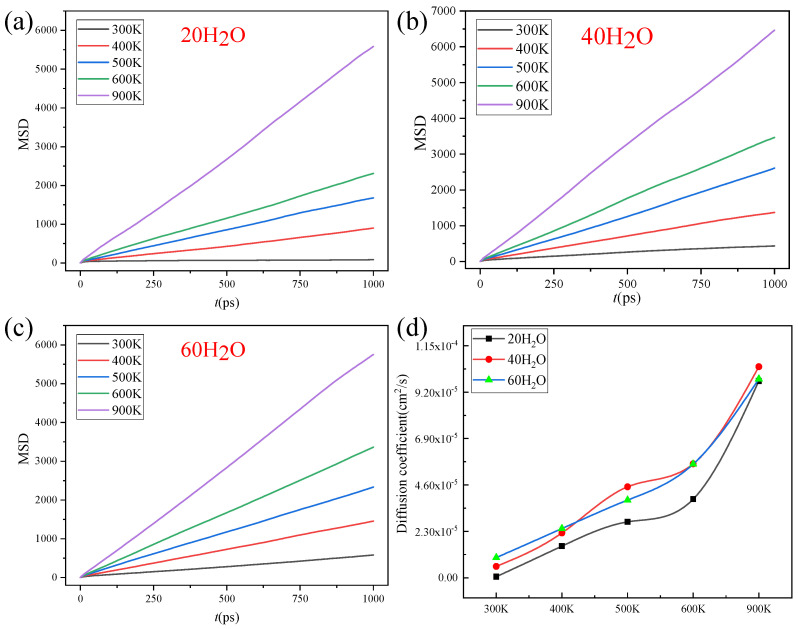
MSD (**a**–**c**) and diffusion coefficient (**d**) of H_2_O on ZIF-90 at different temperatures. (**a**) 20 H_2_O molecules, (**b**) 40 H_2_O molecules, (**c**) 60 H_2_O molecules. (**d**) The diffusion coefficient.

Figure 4 and Figure 5 are the diffusion coefficients of CH_4_ and H_2_O in the ZIF-90 at different temperatures. As can be seen in Figure 4, between 300 K and 400 K, the impact of pressure on the gas diffusion coefficient is minimal. However, when the temperature is above 500 K, the diffusion coefficient of CH_4_ shows a larger change, and at temperatures higher than 600 K, the diffusion coefficient increases rapidly. The maximum diffusion coefficient for 60 CH_4_ is 1.14 × 10^−5^ cm^2^/s, and the minimum is 2.39 × 10^−7^ cm^2^/s. For 40 CH_4_, the maximum diffusion coefficient is 1.25 × 10^−6^ cm^2^/s, and the minimum is 6.28 × 10^−8^ cm^2^/s. For 20 CH_4_, the maximum diffusion coefficient is 2.09 × 10^−6^ cm^2^/s, and the minimum is 9.64 × 10^−9^ cm^2^/s. It can be observed that as the temperature increases, the diffusion coefficient of CH_4_ molecules changes significantly, and with the increase in molecular numbers (the higher the molecular number, the higher the pressure), the diffusion coefficient also shows an increasing trend.

As shown in Figure 5, with the increase in temperature, the diffusion coefficient of H_2_O molecules changes significantly, and as the molecular number (pressure) increases, the diffusion coefficient also increases. The maximum diffusion coefficient for 60 H_2_O is 9.85 × 10^−5^ cm^2^/s, and the minimum is 9.98 × 10^−6^ cm^2^/s. For 40 H_2_O, the maximum diffusion coefficient is 1.04 × 10^−4^ cm^2^/s, and the minimum is 5.64 × 10^−6^ cm^2^/s. For 20 H_2_O, the maximum diffusion coefficient is 9.75 × 10^−5^ cm^2^/s, and the minimum is 4.86 × 10^−7^ cm^2^/s. These data clearly suggest that the diffusion coefficient of water (H_2_O) molecules is significantly higher than that of methane (CH_4_) molecules, by approximately an order of magnitude (×10 cm^2^/s).

Due to the small energy barriers of H_2_O and CH_4_ molecules, the mean square displacement (MSD) and diffusion coefficient increase with increasing temperature. In addition, the influence of gas quantity (pressure) was also explored. For H_2_O molecules, when the number of H_2_O molecules is small, the H_2_O molecules adsorb to aldehyde groups. As the number of H_2_O molecules increases, the diffusion coefficient decreases due to the influence of hydrogen bonds. Therefore, along with the increase in the water (H_2_O) molecule number, the diffusion coefficient of the water (H_2_O) molecules increases first and then decreases. Meanwhile, the adsorption of CH_4_ is weak, so the diffusion coefficient increases with the increase in the number of CH_4_ molecules. Based on the above analysis, the ZIF-90 exhibits diffusion selectivity towards H_2_O/CH_4_ molecules.

### 3.3. Permeation and Separation Performance

**Figure 6:**
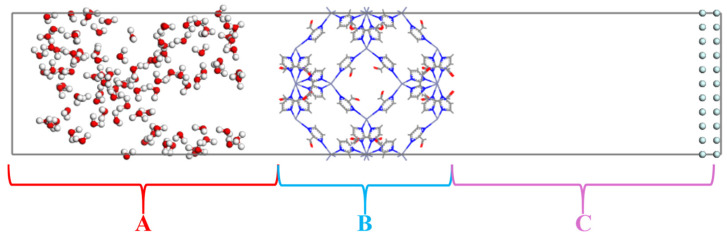
Schematic diagram of the different regions of the ZIF-90.

According to the adsorption and diffusion results of water (H_2_O) as well as methane (CH_4_), the ZIF-90 is an ideal material for the selective separation of water (H_2_O) molecules. To further explore the gas separation selectivity of the ZIF-90, molecular dynamics (MD) simulations were conducted to simulate the permeation of individual molecules and mixtures. The permeation system was divided into three regions (A, B and C), as shown in Figure 6.

**Figure 7:**
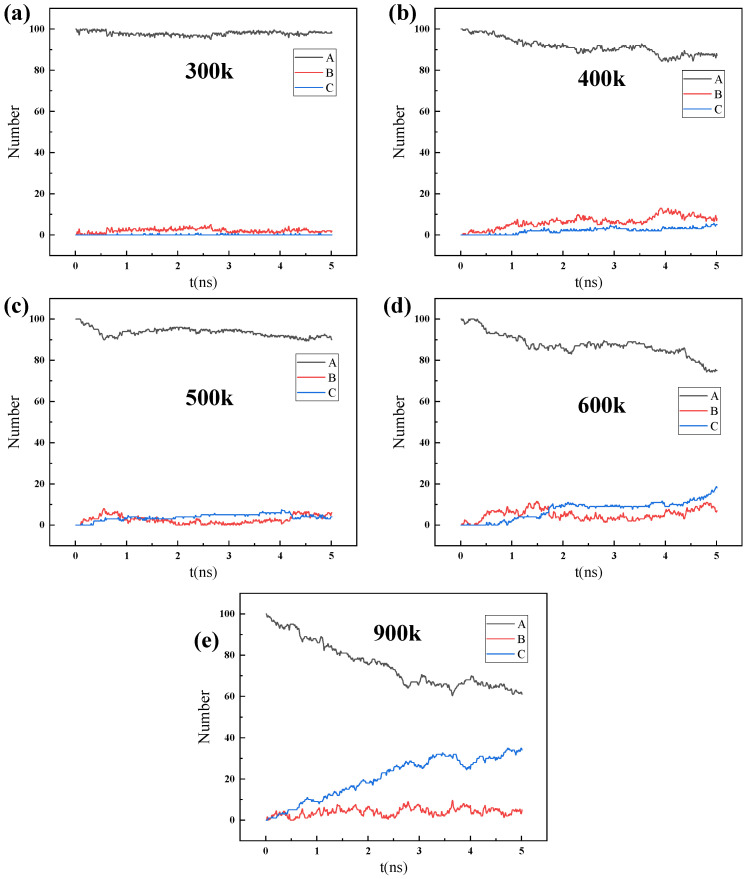
The number of H_2_O molecules in regions A, B, and C changing over time during the process of 100 H_2_O gas molecules permeating through the ZIF-90 membrane at different temperatures: (**a**) 300 K, (**b**) 400 K, (**c**) 500 K, (**d**) 600 K, and (**e**) 900 K.

**Figure 8:**
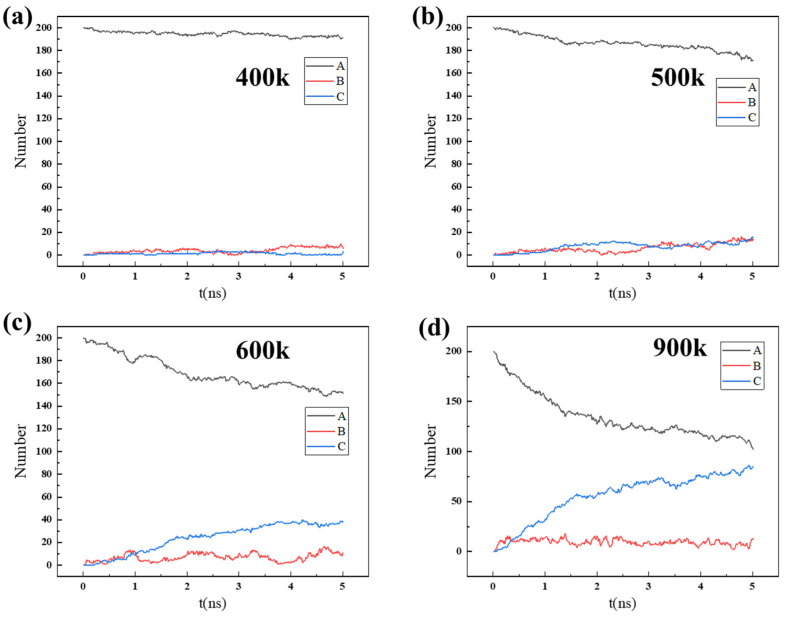
The number of H_2_O molecules in regions A, B, and C changing over time during the process of 200 H_2_O molecules permeating through the ZIF-90 membrane at different temperatures: (**a**) 400 K, (**b**) 500 K, (**c**) 600 K, (**d**) 900 K.

Previous studies [6,25] have confirmed that for ZIF membrane materials, temperature and pressure can affect the adsorption, diffusion, permeation, and separation of water vapor/methane (H_2_O/CH_4_) molecules. Therefore, we investigated the influence of temperature as well as the pressure on the gas separation of ZIF-90 membrane materials, as shown in Figure 7, Figure 8, Figures S1 and S2 (see Supplementary Materials for Figures S1 and S2).

#### 3.3.1. Effects of Temperature and Pressure on Water Molecule Permeation and Separation

Figure S1 (see Supplementary Materials) and Figure 7 are the simulation results of the permeation and separation behavior of 100 water molecules at different temperatures, while Figure S2 and Figure 8 are the simulation results of the permeation and separation behavior of 200 water molecules at different temperatures. In addition, the influence of pressure on the permeation and separation behavior of water molecules was simulated through the difference in the number of water molecules.

For 100 water molecules to permeate, as shown in Figure S1 (see Supplementary Materials), as the temperature increases, the diffusion of water molecules gradually intensifies. At 300 K, the gas molecules have not yet entered region B, indicating that the diffusion of the gas is weak, and it has not yet permeated the ZIF-90. When the temperature is between 300 K and 500 K, the number of water molecules in region A gradually decreases as water molecules enter region B. When the temperature is above 500 K, the number of water molecules in region A continues to decrease, and the water molecules begin to enter region C, indicating the initiation of water molecule separation, which eventually leads to the partial separation of water molecules at 900 K.

In order to further understand the influencing factor of natural gas dehydration, the number of 200 H_2_O molecules in different regions over time was calculated, as shown in Figure S2 (see Supplementary Materials) and Figure 8. For 200 water molecules to permeate, due to the large pressure difference, the diffusion and entry speed of H_2_O gas molecules into the ZIF-90 accelerated. Therefore, the number of H_2_O gas molecules outside the ZIF-90 membrane (in region A) decreases faster over time under higher pressure conditions. The number of H_2_O molecules in region B increases significantly over time. A further analysis of the curves in Figure 8 reveals that H_2_O molecules continuously enter the right side of the ZIF-90 membrane, with the H_2_O molecules filling layer by layer in region C. Previous research [35] results indicate that molecule permeation through pores depends on the adsorption behavior between the molecules and the membrane material, as well as the diffusion energy barrier. The high adsorption energy of H_2_O molecules, coupled with the close proximity to the six-membered rings and the small energy barrier, makes it easier for them to pass through the tunnels.

#### 3.3.2. Effects of Temperature and Pressure on Methane Molecule Permeation and Separation

**Figure 9:**
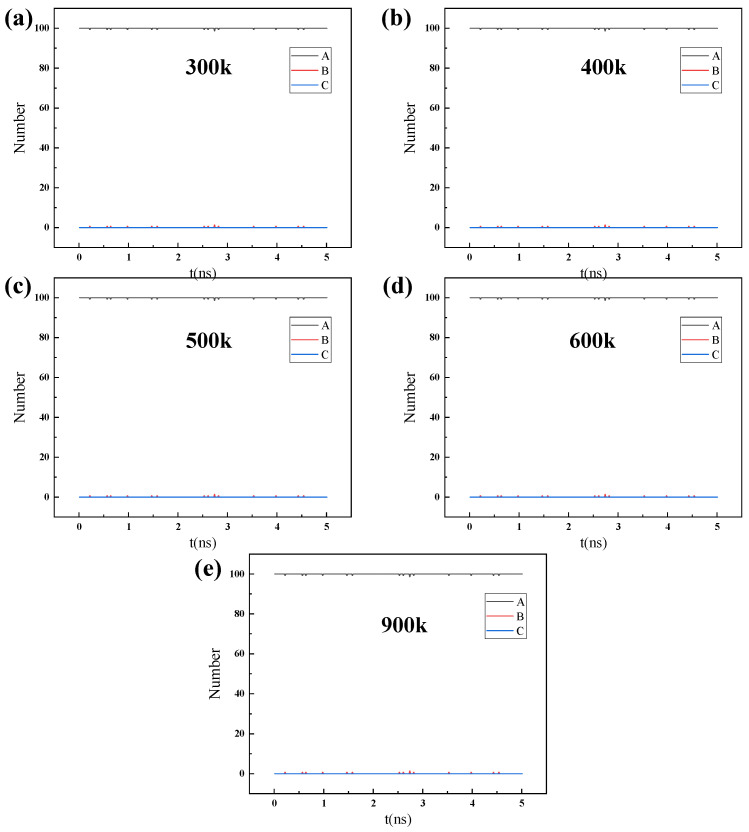
Number of CH_4_ molecules in regions A, B, and C changing over time during the process of 100 CH_4_ gas molecules permeating through the ZIF-90 membrane at different temperatures: (**a**) 300 K, (**b**) 400 K, (**c**) 500 K, (**d**) 600 K, (**e**) 900 K.

**Figure 10:**
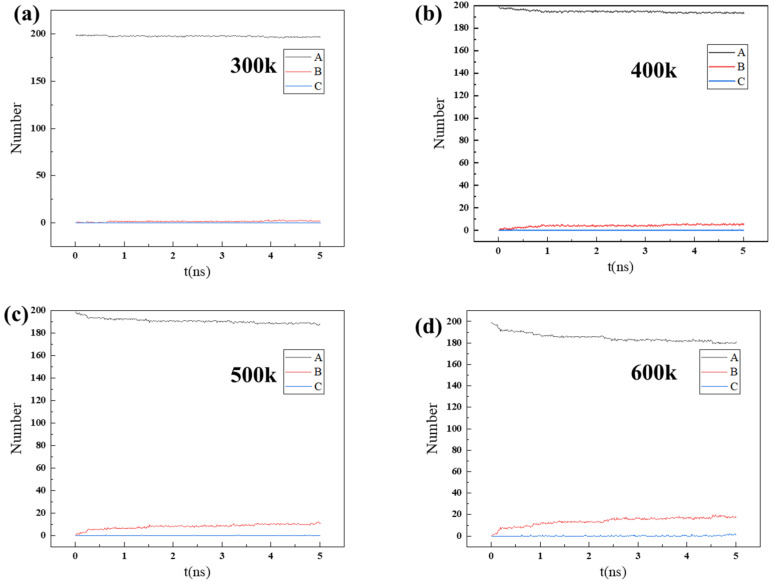
Number of CH_4_ molecules in regions A, B, and C changing over time during the process of 200 CH_4_ gas molecules permeating through the ZIF-90 membrane at different temperatures: (**a**) 300 K, (**b**) 400 K, (**c**) 500 K, (**d**) 600 K.

Figure S3 (see Supplementary Materials) and Figure 9 are the simulation results of the permeation and separation behavior of 100 methane molecules at different temperatures. Figure S4 (see Supplementary Materials) and Figure 10 are the simulation results of the permeation separation behavior of 200 methane molecules at different temperatures. At the same time, the permeability and diffusion behavior of methane molecules in ZIF-90 membrane materials under different pressure conditions was simulated by the difference in the number of methane molecules.

As shown in Figure S3 (see Supplementary Materials) and Figure 9, with 100 methane (CH_4_) molecules, almost no adsorption occurred as the temperature increased. However, as seen in Figure S4, with 200 methane (CH_4_) molecules, the diffusion and adsorption behavior began to appear at 500 K. These results indicate that higher pressure significantly promotes methane (CH_4_) molecule diffusion. Additionally, the calculations of the diffusion energy barrier suggest that methane molecules have a higher diffusion energy barrier compared to water molecules, which is the main reason for the differences in diffusion behavior between water and methane molecules.

#### 3.3.3. Effects of Temperature and Pressure on the Separation of Mixed Gases (CH_4_ and H_2_O) by ZIF-90 Membranes

**Figure 11:**
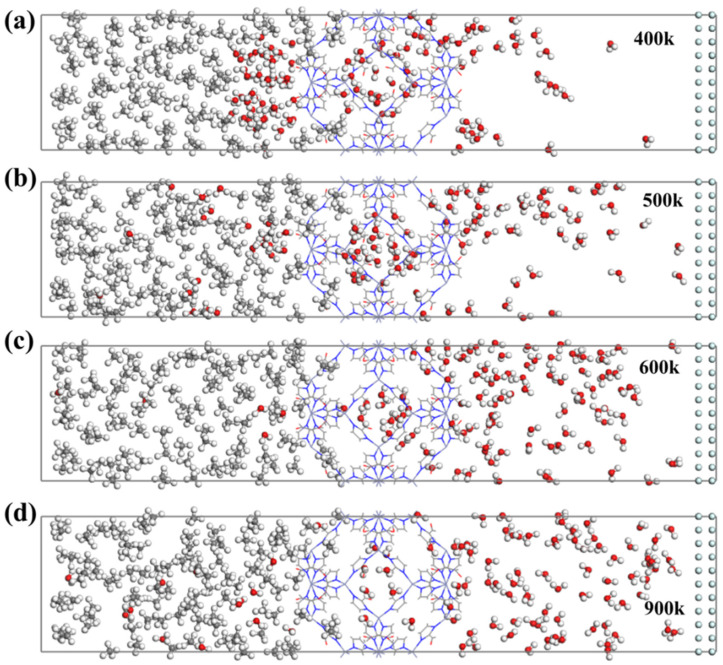
Final state simulation snapshots of the gas mixture (100 H_2_O and 100 CH_4_) permeating through the ZIF-90 membrane at (**a**) 400 K, (**b**) 500 K, (**c**) 600 K, and (**d**) 900 K for 5 ns. (Grey: C; White: H; Red: O).

**Figure 12:**
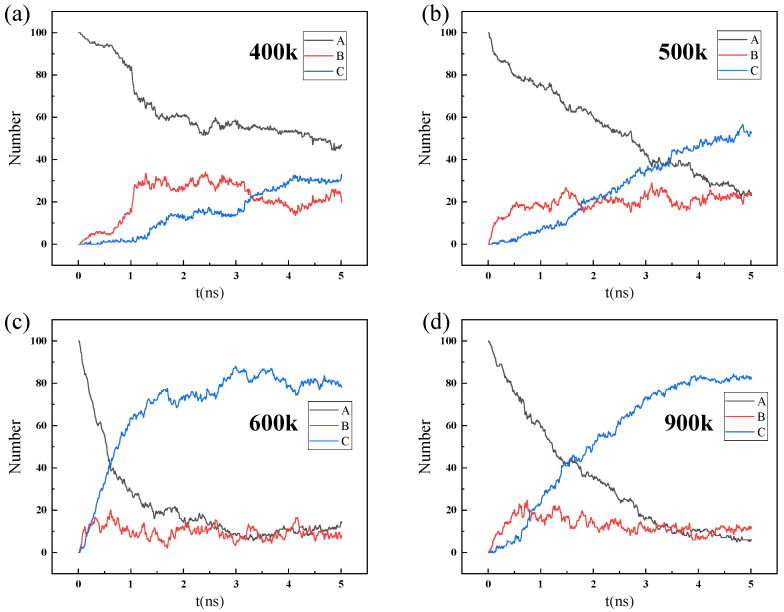
Number of H_2_O molecules in regions A, B, and C changing over time during the process of the mixture gas (100 H_2_O and 100 CH_4_) permeating through the ZIF-90 membrane at different temperatures: (**a**) 400 K, (**b**) 500 K, (**c**) 600 K, (**d**) 900 K.

As shown in Figure 11 and Figure 12, a large number of water (H_2_O) molecules rapidly adsorb and accumulate on the surface of ZIF-90 membranes during the permeation. Under the same conditions, a small amount of CH_4_ molecules adsorb on the surface of ZIF-90 membranes. 

This indicates that the adsorption strength of the ZIF-90 for gas molecules follows the order H_2_O > CH_4_, which is consistent with the computational results from Figure 12. On the other hand, unlike H_2_O molecules, which quickly adsorb onto the ZIF-90 membrane, most CH_4_ molecules cluster and diffuse freely rather than adsorbing on the ZIF-90 membrane due to hydrogen bonding. This suggests that the ZIF-90 enhances the adsorption strength of water molecules. Furthermore, simulation snapshots show that water (H_2_O) molecules can penetrate the ZIF-90 membrane, whereas CH_4_ cannot pass through the ZIF-90 membrane. CH_4_ molecules only enter the first cage and cannot penetrate the second cage. In contrast, H_2_O molecules can easily pass through the ZIF-90 membrane, which indicates that the pore structure of the ZIF-90 membrane plays a crucial role in selectively filtering small gas molecules.

Temperature and pressure significantly affect the permeation process of the mixture gas molecules. Generally, the gas diffusion will accelerate under high temperatures, and a large pressure difference favors gas permeation. The number of water (H_2_O) molecules in region A decreases faster at 900 K compared to 600 K. Due to the rapid gas diffusion behavior at high temperatures, water (H_2_O) molecules are more likely to quickly permeate the ZIF-90 membrane into region C. Similarly, a large pressure difference accelerates the permeation of water (H_2_O) molecules through the ZIF-90 membrane. However, little effect is shown on the permeation of CH_4_ molecules with high temperatures and large pressure differences. The number of molecules entering region B only slightly increases under a high temperature as well as a large pressure difference. Therefore, Zn^+^, as an essential component of the water channel in the ZIF-90 membrane, can effectively block CH_4_ molecules while still allowing H_2_O molecules to permeate through the ZIF-90 membrane with a high temperature and/or a large pressure difference.

The simulation of the permeation of mixed gas molecules of 100 H_2_O molecules and 100 CH_4_ molecules through the ZIF-90 membrane is shown in Figure 11. The snapshots indicate that the surface of the ZIF-90 membrane is covered by H_2_O molecules due to strong adsorption, preventing CH_4_ molecules from approaching and further entering, which is consistent with the density functional theory (DFT) and Monte Carlo simulation of giant regular systems (GCMC) results mentioned above. In the permeation process of simulation, only water (H_2_O) molecules can pass through the ZIF-90 membrane. Compared to the results at lower temperatures, higher temperatures accelerate the diffusion behavior of gas molecules, allowing H_2_O molecules to permeate the membrane more quickly. Furthermore, the pore wall of the ZIF-90 membrane contains polar functional groups, such as aldehyde groups, which makes the membrane hydrophilic. H_2_O molecules can enhance the adsorption and diffusion behavior within the ZIF-90 membrane by forming hydrogen bonds with the polar sites. In contrast, CH_4_ is a non-polar molecule and interacts weakly with the ZIF-90, resulting in a lower adsorption capacity and diffusion rate. Therefore, the hydrophilicity of the ZIF-90 membrane is an important factor leading to its selectivity for H_2_O and CH_4_. Furthermore, the difference in diameter between water molecules and methane molecules mentioned earlier is also a factor that promotes the highly selective penetration of ZIF-90 membranes.

## 4. Conclusions

This paper conducted simulation calculations based on DFT, GCMC, and MD to investigate the process of natural gas dehydration and the effects of temperature as well as pressure. The results are summarized as follows:

1. Simulation calculations based on DFT revealed the mechanisms by which the ZIF-90 separates water from methane molecules. The diffusion energy barriers for the two molecules (CH_4_ and H_2_O) are ΔE(CH_4_) = 155.5 meV and ΔE(H_2_O) = 50.1 meV, respectively.
2. The adsorption isotherms, as well as the diffusion coefficients, were obtained through GCMC and MD simulations, respectively. The results clarify the role of temperature and pressure in the separation of water vapor by the ZIF-90 membrane. Even under high temperature and pressure, the ZIF-90 membrane can separate H_2_O from CH_4_ molecules, efficiently and selectively.
3. The results of natural gas dehydration with the ZIF-90 membrane reveal that Zn^+^ is a crucial component of the water (H_2_O) channel. The Zn^+^ enhances the adsorption of water molecules, reduces the energy barrier for water molecules, and facilitates the natural gas dehydration process with the ZIF-90 membrane.
4. The simulation results elucidate the diffusion mechanisms of water vapor (H_2_O) during natural gas dehydration. The ZIF-90 is more selective of H_2_O, which is mainly due to the strong interaction between the H_2_O molecule and the polar functional groups (such as aldehyde groups) within the ZIF-90.

## Figures and Tables

**Table 1:** Potential parameters and charges of gas molecules.

		ε (eV)	σ (Å)	q
ZIF-90	Zn	0.0071868	3.20	+0.77
	N	0.18415	1.72	−0.403
	C	/	/	−0.105
	H	/	/	+0.213
	O	/	/	−0.367
H_2_O	O	0.0067	3.15	−0.834
	H	0.0020	0.40	+0.417
CH_4_	H	0.00681	3.23	+0.053
	C	0.00233	4.54	−0.212

## Data Availability

All data will be available upon reasonable request.

## Supplementary Materials

The following supporting information can be downloaded at: https://www.mdpi.com/article/10.3390/nano14191583/s1, Figure S1: The final state simulation snapshots of H_2_O molecules permeating through the ZIF-90 membrane at (a) 300 K, (b) 400 K, (c) 500 K, (d) 600 K and (e) 900 K for 5 ns; Figure S2: Final state simulation snapshots of H_2_O molecules permeating through the ZIF-90 membrane at (a) 400 K, (b) 500 K, (c) 600 K and (d) 900 K for 5 ns; Figure S3: Final state simulation snapshots of CH_4_ molecules permeating through the ZIF-90 membrane at (a) 300 K, (b) 400 K, (c) 500 K, (d) 600 K, (e) 900 K temperatures for 5 ns; Figure S4: Final state simulation snapshots of CH_4_ molecules permeating through the ZIF-90 membrane at (a) 300 K, (b) 400 K, (c) 500 K, (d) 600 K for 5 ns.
