# Supplementary material for: Investigation into the Simulation and Mechanisms of Metal–Organic Framework Membrane for Natural Gas Dehydration
Source: Nanomaterials (Basel). 2024 Sep 30;14(19):1583. doi: 10.3390/nano14191583 (PMC11478295; doi:10.3390/nano14191583)
Supplement: Supplementary file 1 [file nanomaterials-14-01583-s001.zip › nanomaterials-3210382-supplementary.pdf]

Supplementary Materials

Investigation into the Simulation and Mechanisms of Metal-Organic Framework Membrane for Natural Gas Dehydration

Qingxiang Song [1], Pengxiao Liu [2], Congjian Zhang [3], Yao Ning [4], Xingjian Pi [4] and Ying Zhang [4,*]

1 College of Science, China University of Petroleum (Beijing), Beijing 102249, China; norm119@163.com

2 PetroChina Tarim Oilfield Company, Kuerle City 841000, China

3 Collaborative Innovation Center of Capital Resource-Recycling Material Technology, College of Materials Science and Engineering, Beijing University of Technology, Beijing 100124, China

4 College of New Energy and Materials, China University of Petroleum (Beijing), Beijing 102249, China

* Correspondence: y.zhang@cup.edu.cn

(1) 100 H_2O molecules

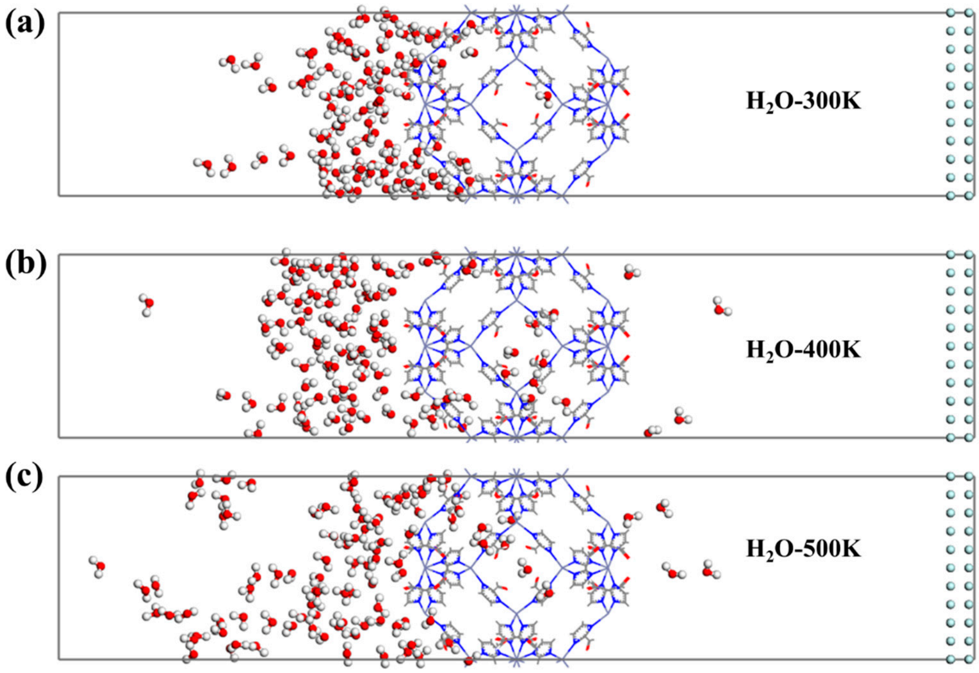

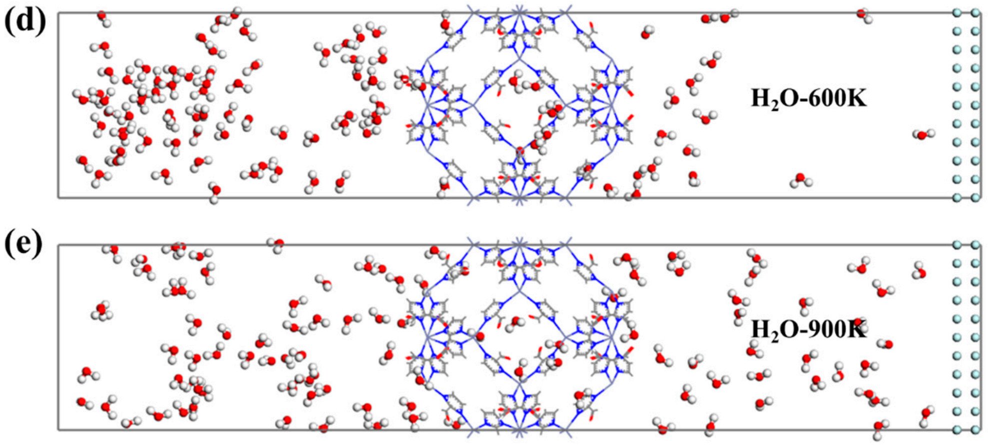

Figure S1. The final state simulation snapshots of H_2O molecules permeating through the ZIF-90 membrane at (a) 300K, (b) 400K, (c) 500K, (d) 600K and (e) 900K for 5 ns.

(2) 200 H_2O molecules

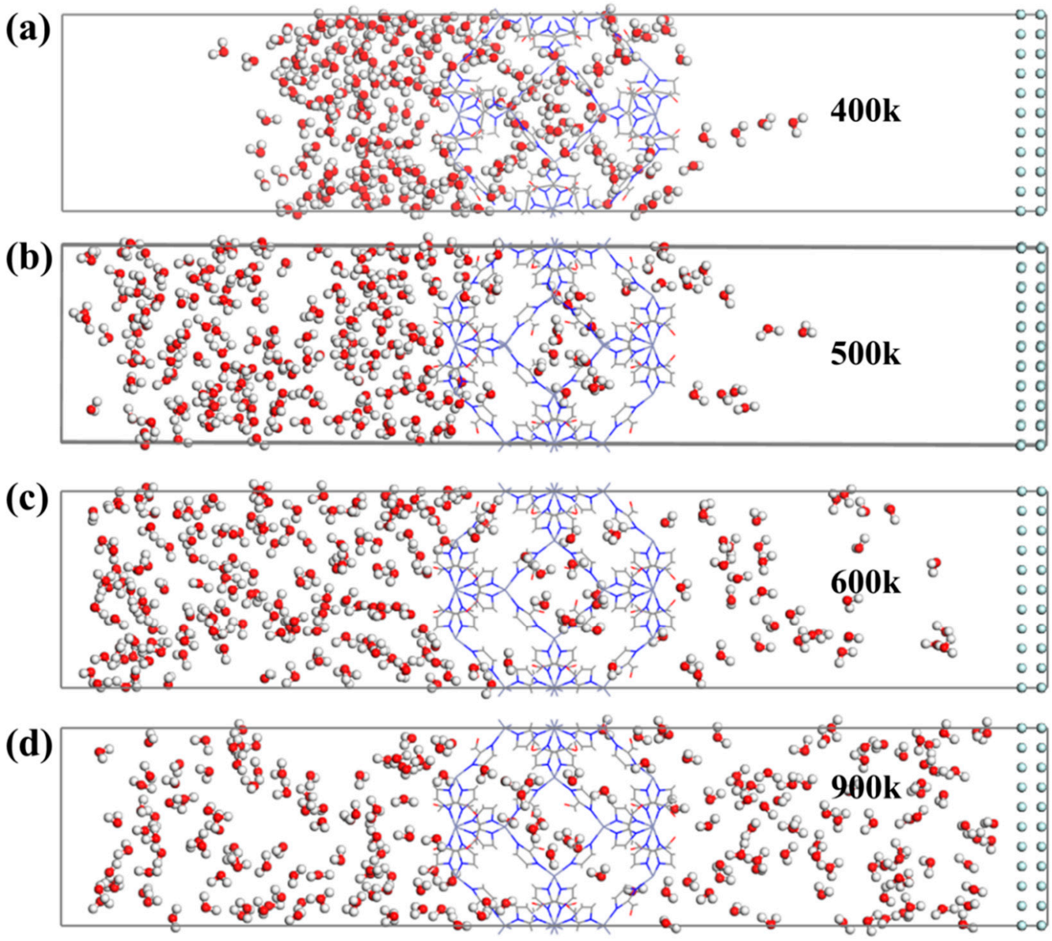

Figure S2. Final state simulation snapshots of H_2O molecules permeating through the ZIF-90 membrane at (a)

400K, (b) 500K, (c) 600K and (d) 900K for 5 ns.

(3) 100 CH_4 molecules

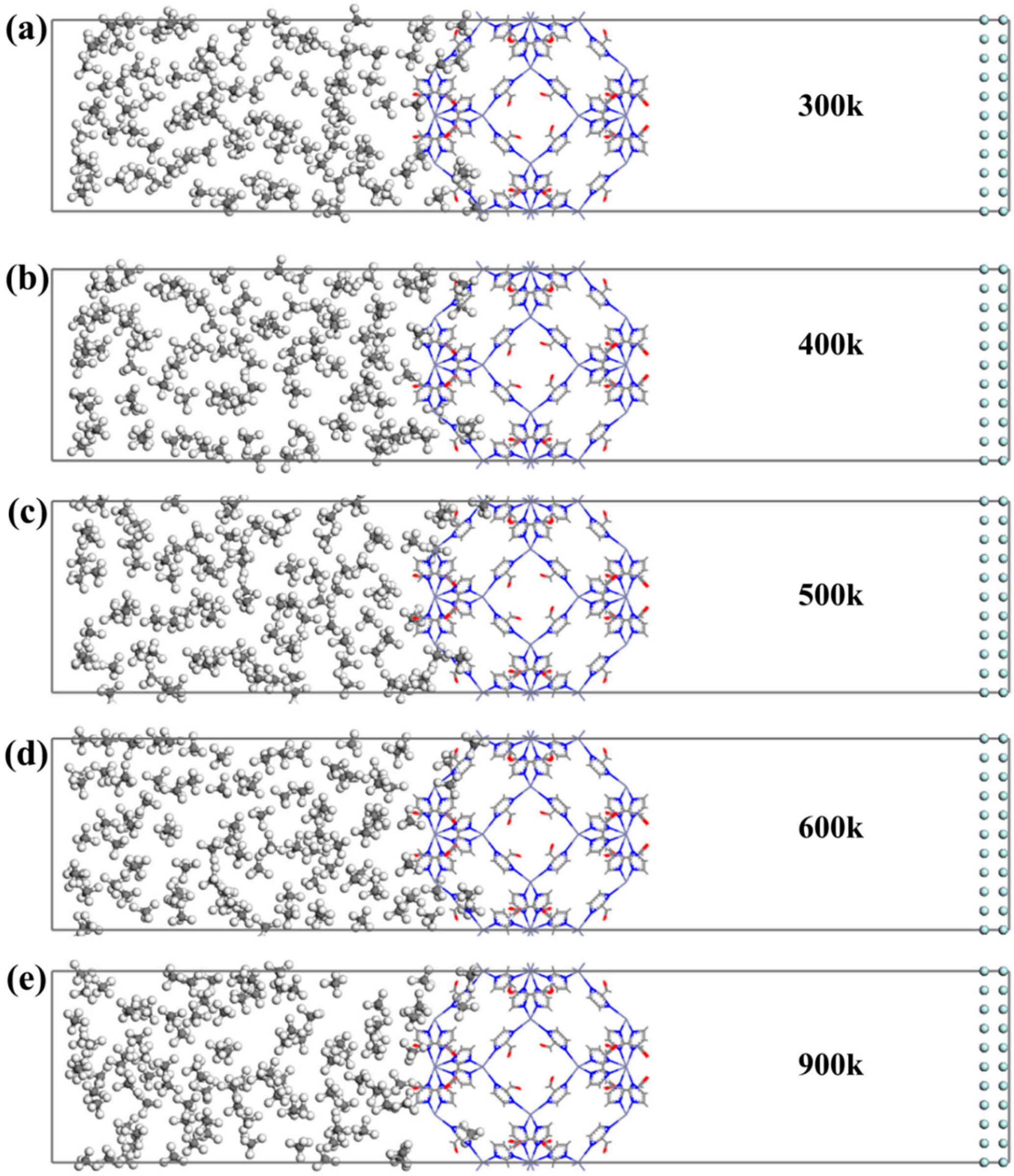

Figure S3. Final state simulation snapshots of CH_4 molecules permeating through the ZIF-90 membrane at (a) 300K, (b) 400K, (c) 500K, (d) 600K, (e) 900K temperatures for 5 ns.

(4) 200 H_2O molecules

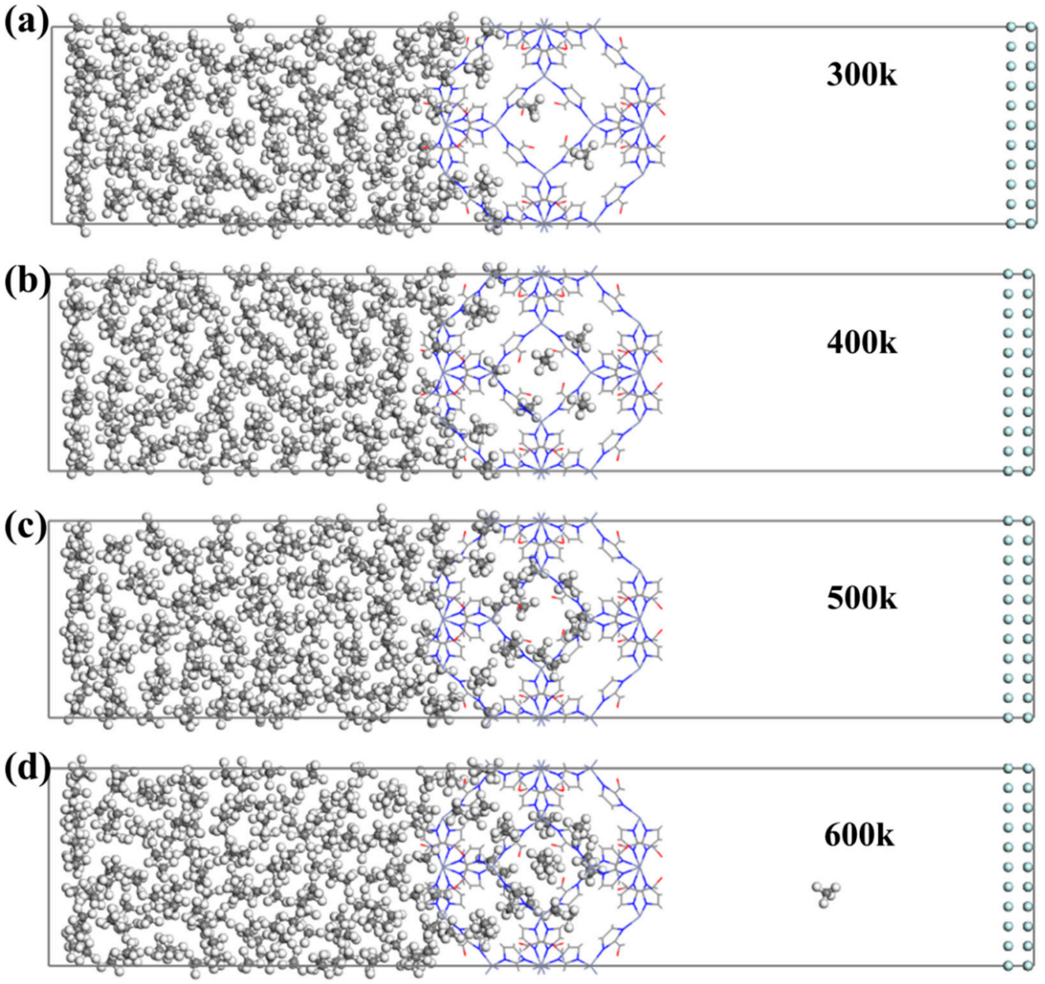

Figure S4. Final state simulation snapshots of CH_4 molecules permeating through the ZIF-90 membrane at (a) 300K, (b) 400K, (c) 500K, (d) 600K for 5 ns.
